# Editorial

**Published:** 1842-04-02

**Authors:** 


### THE MEDICAL EXAMINER.

PHILADELPHIA, APRIL 2, 1842.

### MISCELLANEOUS NOTICES.

*University of Pennsylvania.*—At the annual commencement held on 26th March, the degree of M. D. was conferred on 109 graduates. In July last, 5 received the same degree, making a total for the year of 114.

*Louisville Medical Institute.*—At the fifth annual commencement of this school, held on 3d March, the degree of M. D. was conferred on 53 alumni of the school, and on two graduates from other schools, besides four honorary degrees. The valedictory address was given by Prof. Caldwell. The number of students during the session was 263.

*Transylvania University, at Lexington, Ky.*—The catalogue of this institution shows a list of 271 students during the past session, and of 57 graduates. The valedictory address by Prof. Bartlett.

*Medical College, of the State of South Carolina.*—158 students attended during the past session, of whom 58 received the degree of M. D.

*Medical College of Richmond, Va.*—55 students, 16 graduates.

*Columbia College, at Washington, D. C.*—A list of 20 graduates is published.

*College of Physicians and Surgeons, N. Y.*—25 graduates.

*University of New York.*—51 graduates.

*Willoughby University, Ohio.*—In 1840 and 41, 7 graduates.

*A Board of Naval Surgeons,* composed of Drs. W. P. C. Barton, J. A. Kearney, Thomas Dillard, W. S. W. Ruschenberger, and Waters Smith, will assemble in this city on the 4th inst., for the examination of candidates for the post of assistant surgeons in the Navy, and of assistant surgeons to be passed for promotion.

